# P-1234. Immunoassay showdown: impact on vancomycin-induced kidney injury

**DOI:** 10.1093/ofid/ofaf695.1426

**Published:** 2026-01-11

**Authors:** Joseph Corbino, Martina Boda, Michelle Lee, Emerald O’Rourke

**Affiliations:** Brown University Health, Providence, RI; Brown University Health, Providence, RI; Brown University Health Rhode Island Hospital, Providence, Rhode Island; Brown University Health, Providence, RI

## Abstract

**Background:**

Vancomycin is a glycopeptide antibiotic commonly utilized in empiric and definitive gram-positive infections. Nephrotoxicity is a well-known adverse effect of vancomycin use and is associated with increased length of hospital stay and cost. Risk of nephrotoxicity increases with increasing serum concentrations, with incidence reported up to 37% when troughs are >20 mg/L. Immunoassay is the most common method utilized in clinical practice for measuring serum concentrations due to its cost-effectiveness and ease-of-use, however, lacks the accuracy of complex methods such as chromatography and mass spectrometry. Intraassay variability is described in literature, however data comparing commercially available vancomycin assays in hospitalized patients is scant. This study compares the incidence of acute kidney injury (AKI) in hospitalized patients with two immunoassays, Beckman-Coulter and Abbott Alinity.

**Figure:**
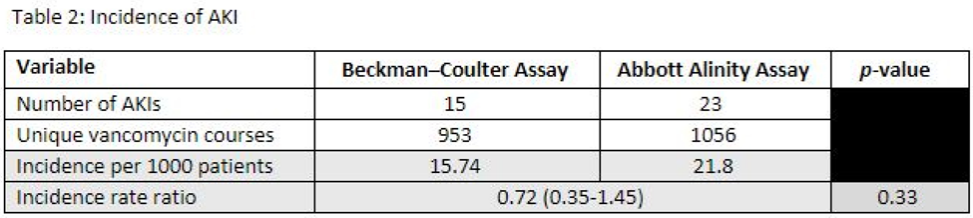

**Methods:**

This retrospective review included adult patients admitted to one of three hospitals in Rhode Island, USA receiving intravenous vancomycin for treatment of a presumed gram-positive infection. The pre- and post-periods were 1 April 2024 – 28 May 2024 (Beckman-Coulter assay) and 11 June 2024 – 8 August 2024 (Abbott Alinity assay), respectively. Patients received vancomycin for ≥48 hours with evidence of AKI within ≤48 hours of vancomycin administration and at least one trough collected prior to AKI onset. Demographic and clinical data were obtained through the electronic medical record.

**Results:**

A total of 38 patients (15 pre-, 23 post-) met inclusion criteria out of all recorded AKIs in the study period. Baseline characteristics were similar between both groups (Table 1). The median time from vancomycin initiation to AKI in the pre- and post- cohort were 5 and 3 days, respectively. The overall incidence of AKI per 1000 patients was 15.7 and 21.8 between the two groups (IRR 0.75, p=0.33). Less than half of the patients experienced resolution of AKI on hospital discharge (46% pre-, 34.8% post-).

**Conclusion:**

Implementation of a new molecular assay did not lead to a statistically or clinically significant reduction in the occurrence of AKIs due to vancomycin. Larger studies are needed to investigate the impact of different immunoassays on incidence of vancomycin-induced AKIs.

**Disclosures:**

All Authors: No reported disclosures

